# More than Just Protein Folding: The Epichaperome, Mastermind of the Cancer Cell

**DOI:** 10.3390/cells14030204

**Published:** 2025-01-30

**Authors:** Haneef Ahmed Amissah, Maxwell Hubert Antwi, Tawfeek Ahmed Amissah, Stephanie E. Combs, Maxim Shevtsov

**Affiliations:** 1 Institute of Life Sciences and Biomedicine, Department of Medical Biology and Biotechnology, School of Medicine and Life Sciences, Far Eastern Federal University, Vladivostok 690922, Russia; haneefamissah@gmail.com; 2 Diagnostics Laboratory Department, Trauma and Specialist Hospital, Winneba CE-122-2486, Central Region, Ghana; 3 Department of Medical Laboratory Science, Faculty of Health and Allied Sciences, Koforidua Technical University, Koforidua EN-112-3991, Eastern Region, Ghana; maxwell.antwi@ktu.edu.gh (M.H.A.); tawfeek.amissah@ktu.edu.gh (T.A.A.); 4 Department of Radiation Oncology, Technische Universität München (TUM), Klinikum Rechts der Isar, 81675 Munich, Germany; stephanie.combs@tum.de; 5 Laboratory of Biomedical Nanotechnologies, Institute of Cytology of the Russian Academy of Sciences (RAS), Saint Petersburg 194064, Russia; 6 Personalized Medicine Centre, Almazov National Medical Research Centre, Saint Petersburg 197341, Russia

**Keywords:** epichaperome, cancer, signaling pathways, liquid–liquid phase separation

## Abstract

The epichaperome, a dynamic and integrated network of chaperone proteins, extends its roles beyond basic protein folding to protein stabilization and intracellular signal transduction to orchestrating a multitude of cellular processes critical for tumor survival. In this review, we explore the multifaceted roles of the epichaperome, delving into its diverse cellular locations, factors that modulate its formation and function, its liquid–liquid phase separation, and the key signaling and crosstalk pathways it regulates, including cellular metabolism and intracellular signal transduction. We further highlight techniques for isolating and identifying epichaperome networks, pitfalls, and opportunities. Further, we review the profound implications of the epichaperome for cancer treatment and therapy design, underscoring the need for strategic engineering that hinges on a comprehensive insight into the comprehensive structure and workings of the epichaperome across the heterogeneous cell subpopulations in the tumor milieu. By presenting a holistic view of the epichaperome’s functions and mechanisms, we aim to underscore its potential as a key target for novel anti-cancer strategies, revealing that the epichaperome is not merely a piece of protein folding machinery but a mastermind that facilitates the malignant phenotype.

## 1. Introduction

The cellular landscape is highly dependent on the complex interplay of molecular players that dictate cell fate, proliferation, metabolism, and, ultimately, survival [1]. Within this complex milieu, molecular chaperones have traditionally been viewed in the premise of cellular “helpers” that interact with their target proteins and aid in their protein folding and the related processes of proteostasis [2,3]. But then, recent evidence has shown that molecular chaperones interact in a complex, highly interconnected network that includes co-chaperones, adapter proteins, isomerases, and foldases [4,5].

While these structural compositions and functions of molecular chaperones remain critical and have been widely accepted in the scientific community, an emerging concept of an interactome characteristic of cancer, the “epichaperome”, has emerged and is gaining ground within the scientific community [6], the discovery of which has been dependent on advances in proteomics, advanced biomedical instrumentation, systems biology, and bioinformatics that have surmounted the difficulties of processing large data sets, enabling complex interactome analysis.

Contrasting with molecular chaperones, which are transient and intrinsically dynamic and exist in both physiological and pathological conditions, the epichaperome is a stable and perpetual hetero-oligomeric chaperone network bounded by cofactors that are present exclusively in pathological states, like cancer. The epichaperome is spatiotemporally organized with diverse locations in specific subcellular compartments [7]. In these locations, the epichaperome acts as a dysfunctional, long-lived scaffold assembly of chaperones that stabilize and promote protein folding and maintains aberrant proteome-wide interactions [8,9], which exacerbates disease pathogenesis.

Hence, an in-depth introspection into the specific constituents in diverse cellular situations remains a critical challenge of epichaperome biology yet to be fully elucidated. Indeed, the epichaperome exerts control over various aspects of cellular function, including but not limited to intracellular signal transduction, autophagy, and nuclear dynamics (gene regulation, chromatin/nucleosome remodeling, transcriptional factor chaperoning, global chromatin changes, genome replication, and the transcription of genes), which makes it a central hub to promote the dysregulated cellular processes seen in cancer cells, promoting their survival and relentless growth.

This review provides an overview of the evolving landscape of epichaperome research, emphasizing its dynamic presence across various cellular compartments. We highlight the diverse functions of the epichaperome in regulating cellular processes by hijacking and regulating intracellular signal transduction that allows the cancer cell to respond to different stimuli. We also delve into the mechanisms governing the epichaperome, including its transcriptional control, the interplay of co-chaperones, and the impact of its ATPase activity. Additionally, we discuss the emerging concept of the liquid–liquid phase separation of the epichaperome in the cancer cell while addressing the need for advanced technologies to better study and characterize dynamic interactomes such as the epichaperome. We also highlight the therapeutic implications of these findings, exploring the potential of targeting the epichaperome for cancer treatment and innovative therapy design.

This comprehensive review underscores the crucial role of the epichaperome in cellular biology and its significance as a target for future cancer interventions.

## 2. The Biology of Epichaperomes and Interactomes

The epichaperome, with its multicomponent network, goes beyond protein folding to regulate diverse cellular functions. This section delves into how this network of chaperones is deployed within cancer cells, its regulation, its connections to canonical signaling pathways, and its impact on metabolic processes that fuel cancer cell survival and growth.

### 2.1. Cellular Locations of Epichaperome and Epichaperome Constituents

The optimal functioning of a system demands the effective operation of its constituent structures, substructures, and functional elements, analogous to the cell as a system. For a cell, a prerequisite to fulfilling these requirements is the proper functioning and a well-coordinated homeostatic balance in its “workhorses” in all structural and substructural compartments [10,11]. To meet these demands, cells invest in molecular chaperones to optimize the efficiency of these processes in membrane-bounded cytosol and organelles, as outlined in Table 1.

Responding to the range of cellular demands, molecular chaperones deploy and function in diverse, multicomponent networks that operate in different cellular compartments such as the endoplasmic reticulum/Golgi apparatus [12,13,14], nucleus [15], mitochondria [16], and the intra- and extracellular compartments of the cell membrane [17,18], including secreted forms in the extracellular space [19,20] and the cytosol [21].

The cytosol has been the primary focus of most research on protein homeostasis. The cytosol is known to house constitutively expressed levels of heat shock protein 110 (Hsp110), Hsp90, Hsp70, and Hsp60 and the nucleotide exchange factor small molecular chaperones, the Hsp40 family [21]. Within these groups of chaperones, Hsp110, Hsp90, Hsp70, and Hsp60 are known to bind and use ATP. These molecular chaperones interact with one another within a complex network. These interactions are mediated via co-chaperones and chaperonins. Important for the proper function of the network is Hsp70’s interaction with Hsp40, which facilitates the delivery of client proteins to Hsp70. Together, these protein–protein interactions (PPIs) stabilize misfolded, metastable, and non-native proteins, providing conditions for refolding while promoting the proteolysis of ubiquitinated proteins.

In the endoplasmic reticulum (ER) and the Golgi apparatus, the analogous form of Hsp70 (BiP) [12] in concert with GRP170 (Hsp110 homolog) [22], Sil1 [23], ERdj3/Sec63 [24,25], and a host of other chaperones, aids the unilateral ER luminal translocation of co- and post-translational proteins. These proteins are translocated through the Sec61 channels and held in soluble conformations in the ER lumen, where they are folded into their 3-D structure or undergo endoplasmic reticulum-associated protein degradation (ERAD), depending on their structural conformation or the cell’s functional state [26]. Facilitating 3-D folding, thiol oxidoreductases like protein disulfide isomerase create the protein disulfide bonds crucial for proper protein folding [27,28]. Additionally, lectin-like chaperones such as calnexin and calreticulin can act as a scaffold to recruit function-specific ER chaperones and can also potentiate thiol oxidoreductases [29] to facilitate the formation of disulfide bonds, proline isomerization and the structural maturation of proteins [30]. In response to stress situations, calnexin and calreticulin can signal the unfolded protein response (UPR) pathway [29]. Likewise, the ER Hsp90 analog GRP94 [31] participates in protein folding and interacts with ER protein folding machinery [32] to regulate Ca^2+^ homeostasis and targets misfolded proteins for ERAD in response to stress. In a coordinated fashion, ER and Golgi apparatus chaperone networks crosstalk to effectively regulate protein homeostasis activities [13].

In the mitochondria, most of the workhorses requisite for its function are synthesized from the mitochondrial genome. However, many of its other needs are dependent on proteins encoded in the nuclear genome. Thus, these extramitochondrial proteins require translocation from the ER through the cytoplasm to the mitochondria. Here, in organello import assays have proven that post-translationally modified proteins can translocate into the mitochondria [33,34]. The mitochondrial Hsp70 (mtHsp70/GRP75/mortalin), with “unfoldase” activity, multinetworks with other chaperones to form complexes crucial for mitochondrial protein homeostasis [35]. In a related study, Böttinger et al. demonstrated that Hsp60, when aided by its cofactor Hsp10, folds some of mtHsp70’s client proteins [35]. However, the functionality of Hsp60 is dependent on the binding of its cofactor to mtHsp70, forming an mtHsp70/Hsp60/Hsp10 complex that activates Hsp60 and in turn modifies the functional specificity of mtHsp70 [35]. The mtHsp70/Hsp60/Hsp10 complex can interact with the mitochondrial Hsp90 paralog, TNF-associated protein 1 (TRAP1), and has been noted to play a role in the regulation of oxidative phosphorylation in the mitochondria [36]. Extramitochondrial proteins from the nuclear genome are shuttled into the mitochondria by an mtHsp70/Tim44 (the peripheral subunit of the Tim23 complex)/Mge1 presequence translocase-associated motor (PAM) machinery in an ATPase-dependent reaction cycle [37]. MtHsp70 then interacts with DNAJA3 in a Lon protease (LONP1)-dependent fashion to provide a stable environment for precursor protein folding [38].

Moreover, Hsps play an equally crucial role in nuclear processes, including gene regulation, chromatin/nucleosome remodeling, transcriptional factor chaperoning, global chromatin changes, genome replication, and the transcription of genes [39]. Using YK5-B bait, the epichaperome Hsp70 (epiHsp70) has been demonstrated to interact with an array of proteins, including the Nuclear Mitotic Apparatus Protein 1 (NUMA1), which is involved in the formation and maintenance of spindle poles and kinetochore alignments for sister chromatid segregation during mitosis [40]. Recently, a study by Xu et al. highlighted the regulatory role of Hsp90 in cytokinesis [41]. Hsp90, through interactions with its nuclear co-chaperone NudC-like protein 2 (NudCL2) [42], regulates cytokinesis by stabilizing the regulator of chromosome condensation 2 (RCC2) at the midbody of mammalian cells. As such, RCC2 and/or NudCL2 downregulation results in cytokinesis failure, the multinucleation of daughter cells, and midbody disorganization. NudCL2 is also noted to interact with Hsp70, suggesting a possible Hsp70/Hsp90/NudCL2 triage where Hsp70 could modulate the clientele of the nuclear Hsp90 (nHsp90) [5]. Moreover, Hsp90 also binds and aids the nuclear shuttling of HDAC3 to regulate histone modification processes and gene expression patterns [43]. The systematic investigation of RNA-binding chaperones reveals some intriguing insights. Intriguingly, it has been found that at least one member from each of the Hsp families can bind RNA, revealing an unexpected functional diversity among these proteins. In global chromatin profiling, the Sawarkar group has shown that HSPA1A, the Hsp70 nuclear homolog, binds and interacts with non-coding RNAs and can occupy and regulate RNA Polymerase III activity at its active transcribed genomic loci by inhibiting nascent tRNA transcripts [44].

We now also know that molecular chaperones exist in the extracellular space as well as on the extracellular membrane and on the intracellular compartment of the cell membrane. For instance, Hsp70 has been reported to show a predilection towards negatively charged phosphatidylserine (PS)-composed membranes [45]. In our recent work, we have demonstrated that Hsp70 binds to the cell membrane and is associated with high tumor cell migration activity [17]. Also, in melanoma cells, Hsp90 is reported to be expressed in the extracellular compartment of the cell membrane [46]. Amphitropic small Hsps also show interactions with cell membranes, though they lack transmembrane domains or signal sequences [18]. Extracellular forms of Hsp90 linked to the activation of MMP-2 on fibrosarcoma cells [19] act by interacting with Hsp70, Hsp40, Hop, p23, and Hip in an ATP-independent manner [20]. It is our view that the presence of these chaperones in and on the cell membrane and extracellular space is vital for maintaining membrane and secreted extracellular proteins in their active 3-D state for proper cellular functioning and signaling such as immune activation, cytokine signaling, angiogenesis, extracellular matrix remodeling, etc.

It is thus important to note that molecular chaperones in the various cellular compartments regulate many bioprocesses, including protein folding, PTM and metabolism, actin dynamics and morphogenesis, global chromatin and gene regulation, mitosis, vesicle trafficking, and ribosomal and RNA dynamics, amongst others, by forming dynamic and intricate network systems [47]. The efficiency of dispensing these roles is dependent on a “collective labor” approach in the network while crosstalking with networks in other cellular compartments in a coordinated fashion to meet the demands of various bioprocesses. Thus, under cellular stress, particularly in cancer, achieving adequate cellular functioning and survival necessitates a well-orchestrated cellular system. This requires concerted efforts of the protein machinery across all cellular compartments to manage the excessive proteostasis demands in this state. This observation leads one to speculate an epichaperome-like coordination amongst the homolog forms of Hsp70 and Hsp90, both intracellularly and extracellularly, that ensures the synchronization of the cell’s protein demands and diverse coordinated roles of the epichaperome. Another possibility is that the cytosolic epichaperome network coordinates the activities of all other chaperone networks in the different cellular locations.

Supposing that these hypotheses hold, this raises some questions worth exploring. How do the networks in other cellular compartments mentioned above interconnect? Could the cytosolic epichaperome be the mastermind of these dynamics, and if yes, how?

**Table 1:** Cellular locations of epichaperome constituents and their roles in cellular function.

Cellular Location	Molecular Chaperone(s)	Role	Reference
Cytosol	Hsp90	The nucleation and signaling hub of the multichaperone network that acts in the maturation stages of client protein folding.	[21]
	Hso70/Hsp40 (nucleotide exchange factor [NEF])	1. Supports, shuffles, and loads client proteins into Hsp90 with the support of Hsp40 family proteins. 2. Partakes in protein folding, disaggregates aggregates, and can translocate to other membrane-bound cellular compartments to support chaperoning activity. 3. Plays a role in chaperone-mediated autophagy.	[21]
	Hsp110, Hsp90, Hsp70, Hsp60, and Hsp40 complex	Interacts in a complex loop mechanism with support from co-chaperones to chaperone-misfolded, metastable, and non-native proteins, providing suitable conditions for refolding while promoting the proteolysis of ubiquitinated proteins that culminates in cellular proteostasis.	[21]
ER/Golgi apparatus	BiP (Hsp70 homolog)/GRP170 (Hsp110 homologue)/Sil (ER-NEF)/ERdj3 (Hsp40 Homolog)/Sec63 (transmembrane protein)	1. Aid the unilateral ER luminal translocation of co- and post-translational protein. 2. Hold ER-translocated proteins in soluble conformations and fold them into their 3-D structure or activate ERAD with the assistance of GRP94.	[12,22,23,24,25,26]
	Protein disulfide isomerases (thiol oxidoreductases)	Facilitates the 3-D folding of ER polypeptides by creating the protein disulfide bonds crucial for proper protein folding.	[27,28]
	Calnexin and calreticulin	1. Acts as a scaffold to recruit function-specific ER chaperones. 2. Potentiates thiol oxidoreductases to facilitate the formation of disulfide bonds, proline isomerization, and the structural maturation of proteins. 3. Coordinates the signaling of UPR in stress conditions.	[29,30]
	GRP94 (Hsp90 homolog)	1. Participates in ER protein folding. 2. Interacts with ER protein folding machinery to regulate Ca^2+^ homeostasis. 3. Targets misfolded proteins for ERAD in response to stress.	[31,32]
Mitochondria	mtHsp70/GRP75/mortalin (Hsp70 homolog)	1. Has “unfoldase” activity that allows for the disaggregation of aggregated proteins/peptides. 2. Multinetwork with other chaperones to form complexes crucial for mitochondrial protein homeostasis.	[35]
	mtHsp70/Hsp60/Hsp10	1. mtHsp70 interacts with Hsp10, which is needed for Hsp60 activity to form the mtHsp70/Hsp60/Hsp10 complex that modifies the functional specificity of mtHsp70. 2. The MtHsp70/Hsp60/Hsp10 complex interacts with TNF-associated protein 1 (TRAP1) (Hsp90 homolog) to regulate oxidative phosphorylation in the mitochondria.	[35,36]
	mtHsp70/Tim44 (peripheral subunit of the Tim23 complex)	Translocate extramitochondrial proteins through the presequence translocase-associated motor (PAM) machinery in an ATPase-dependent reaction cycle.	[37]
	mtHsp70/DNAJA3 (Hsp40 family protein)/LONP1	Provide a stable environment for precursor protein folding.	[29]
Nucleus	Diverse chaperone complexes in the nucleus	Crucial for roles including gene regulation, chromatin/nucleosome remodeling, transcriptional factor chaperoning, global chromatin changes, genome replication, and gene transcription.	[39]
	epiHsp70/NUMA1	Crucial for the formation and maintenance of spindle poles and kinetochore alignments for sister chromatid segregation during mitosis.	[31]
	nHsp90/nHsp70/NudCL2/RCC2	Regulates cytokinesis by interacting with its nuclear co-chaperone, NudCL2, to stabilize the regulator of chromosome condensation 2 (RCC2) at the midbody of mammalian cells.	[41,42]
	nHsp90/HDAC	Regulates histone modification processes and gene expression patterns.	[43]
	HSPA1A (Hsp70 nuclear homolog)	Interacts with non-coding RNAs and can occupy and regulate RNA Polymerase III activity at its active transcribed genomic loci by inhibiting nascent tRNA transcripts.	[36]
Extracellular/membrane-bound forms	Membrane-bound Hsp70 (mHsp70)	Shows a predilection for negatively charged phosphatidylserine (PS)-composed membranes and chaperone membrane proteins involved in membrane actin interactions for cell motility, invasion, and cellular protection from various stresses.	[17,45,48]
	mHsp90	1. Expressed on the extracellular membrane compartment of melanoma cells and is associated with tumor progression.	[46]
		2. Interacts with Hsp70, Hsp40, Hop, p23, and Hip in an ATP-independent manner in the extracellular space to activate MMP-2 on fibrosarcoma cells.	[19,20]

### 2.2. Epichaperome Formation in Tumor Cells

Unraveling the epichaperome’s architecture is crucial to elucidating the key players and their facilitating “helpers”, which drives a better understanding of the functionality of this intricate system. Insights into the functional domains of each member and their collaborative integration have been the pivot upon which functional inhibitors of the epichaperome have been pieced together. Yet, up until now, the precise assembly of this complex network has remained largely uncharacterized. In this section, we describe how the epichaperome scaffolding within cancer cells forms, beginning with an overview of molecular chaperones in normal cells.

Like all native proteins, chaperone interactions unfold through a dynamic series of hydrogen, hydrophobic, electrostatic, and van der Waals interactions [49]. Within healthy cells, chaperones organize into pockets of functional networks. The most abundant molecular chaperone, Hsp70, does not act alone but interacts with partners such as the co-chaperone Hsp40 and the nucleotide exchange factors (NEFs) (Bcl-2-associated anthanogene domain proteins [50]). Together, this complex facilitates and regulates the Hsp70 folding of denatured client proteins (including neuroblast differentiation-associated protein, Filamin-A, clathrin heavy chain 1, etc.) [51] by recognizing the short hydrophobic peptide regions of client proteins in an ATP-dependent manner [52].

In the Hsp70 machinery, Hsp40 plays a crucial role, acting as the primary recruiter of client proteins. Hsp40 acts by binding client proteins through its helical J-domain while simultaneously stimulating Hsp70 ATPase activity [53]. Thus, Hsp40 effectively captures and holds unfolded or misfolded proteins and delivers them to Hsp70 for the final protein folding. This handoff process is supported by the NEFs (Hsp110), which regulate the Hsp70 cycle [54] and ultimately ensure the proper conformation of misfolded client proteins, preventing the accumulation of potentially harmful protein aggregates.

Hsp90, another abundant molecular chaperone, exists as a dimer (the inducible Hsp90α form and the constitutive Hsp90β form), with each dimer composed of a C-terminal, middle domain, and the N-terminal domains [55,56]. The functional reach of Hsp90 expands through its interactions with crucial co-chaperones. Of note, Cdc37, for instance, binds Hsp90, identifies its client kinase proteins, and loads them onto Hsp90 [57]. Also, the immunophilin FK506-binding protein (FKBP52) [58] is indispensable for steroid receptor maturation. Both Cdc37 and FKBP52 utilize conserved tetratricopeptide domains (TRPs) within the C-terminal motif of Hsp90 to interact with it. Also, this common TRP domain also enables the Hsp90-Hsp70 organizing protein (Hop) to promote their linkage [59], facilitating efficient substrate delivery to Hsp90 [60]. Complementing the activities of the TRP-domain-containing co-chaperones are Aha1, the Hsp90 ATPase regulator, and p23, which facilitate the ATP cycle of Hsp90 client protein binding to modulate client protein maturation [61,62].

Additional to the Hsp70 and Hsp90 machinery is the Hsp60 molecular chaperone pocket, which is broadly categorized into two groups (I and II). Group I identified in *E. coli* as GroEL and co-chaperones with GroES also includes the mitochondria and chloroplast forms. In contrast, the group II chaperonins seen in archaea and found in eukaryotes are known as chaperonin-containing tailless complex polypeptide-1 (TCP1)/TriC/CCT [21]. Hsp60’s influence extends throughout the cell but is primarily localized within the mitochondria, cytosol, and cell membrane [63]. Together with Hsp70, they constitute the main chaperone machinery within cells [64]. Distinctly, Hsp60 is made up of two oligomeric rings, each composed of eight homologous subunits, where client proteins (like actin and tubulin) undergo assisted folding [65]. This process is dependent on ATP hydrolysis, coupled with interactions with Hsp10, which influences its distinct tertiary structure formation to potentiate its activity [66].

The final pocket is the Hsp100/caseinolytic protease family (Clp) (AAA+ chaperones) and the small heat shock proteins (sHsps). Hsp100 chaperones act as disaggregases and unfoldases [67], which deliver misfolded and aggregated proteins for refolding. While Hsp100 is known for its disaggregase and unfoldase activity, it is also known that Hsp100 can, in some cases, induce protein degradation by forming a barrel-like peptidase structure with the Clp group, such as ClpP protease, to degrade aggregated proteins [68]. Largely, resolubilized proteins are passed on to Hsp70 and Hsp60 machinery for their subsequent refolding [69]. In different organelles, Hsp100 harnesses ATP to mechanically unwind or thread polypeptides by passing them through their central channel, rescuing proteins from stress-induced misfolding [21].

In contrast, the sHsps represent a different strategy. These ATP-independent chaperones are characterized by their α-crystallin domain, specialized in binding and stabilizing partially folded or intermediate proteins, preventing them from misfolding or aggregating [70]. It is noteworthy to indicate that sHsps act very early in the chaperone folding cycle. together with the Hsp100 chaperones, after resolubilizing misfolded or aggregated proteins by preventing their irreversible folding [70]. With their disordered and flexible N- and C-termini, sHsps readily interact with exposed hydrophobic surface residues of intermediate folded proteins as well as with other sHsps [70].

To summarize, these distinct molecular chaperones play specific roles: Hsp100 acts as an unfoldase and disaggregase to resolubilize misfolded or aggregated proteins for their spontaneous refolding; sHsps act as holdases, preventing the misfolding of partially folded or resolubilized proteins; and Hsp90, Hsp70, and Hsp60 act as foldases to promote the proper 3D folding of soluble proteins [71]. It is important to stress at this point that the interactions between these chaperone complexes in normal cells are transient and sparing and occur in a precisely controlled fashion [6,72].

Following stress conditions, such as heat shock, normal cells trigger the heat shock response (HSR). HSR reprograms the cell’s gene and enhancer network by upregulating, among others, the heat shock factor 1 (*HSF1*) gene that promotes the transcription of hsp synthesis [2,73]. Accordingly, the cell reprograms its internal programs, including a tight coordination of its proteostasis network, orchestrating cell survival. Here, the respective hsp pockets discussed above intensify their activity to stabilize the cell by redistributing its proteostasis activity in a “loading shedding” system. Hence, less burdened chaperone pockets compensate for those overwhelmed through transient low interaction strengths [6]. As the stress diminishes, so does the cell reprogram its gene and enhancer networks, returning to basal levels. However, it should be noted that these dynamics play out in acute stress conditions.

In cancer cells, a different reality unfolds. While acute stress prompts a transient chaperone response, chronic stress, the obvious factor in the tumor microenvironment, dramatically reshapes the cellular landscape. Aneuploidy, and the resulting altered protein stoichiometries, alters gene networks and enhancer networks in a distinct way. Here, some cancer cells forge a strong, stable, hyperconnected chaperome network, the epichaperome. This hyperconnected network incorporates scaffold proteins, interface modulator proteins, and adaptor proteins (“connectors”) to connect all chaperome pockets into a single functional pocket capable of modulating diverse cellular functions. Epichaperome hyperconnectivity is reinforced by the downstream effect of the *MYC* gene, ensuring the cancer cell’s survival [6]. The reprogramming phase shifts the epichaperome’s thermodynamic state, fostering stronger, more persistent interactions between all the chaperone hubs—contrasting with the weaker, transient bonds and insular Hsp90 and Hsp70-only network connected by Hop as seen in acute stress and in normal cells [6]. The epichaperome origin begins with Hsp90 and Hsp70, acting as nucleation sites. These hubs then draw in other chaperone pockets (hubs) through “connectors,” thereby modulating the structural stability and functionality of the chaperone pockets to handle cancer cell-specific cellular needs by rewiring old networks and formulating new networks in a context-based manner by spontaneous response to fluctuating molecular cues linked to stressors contingent for survival.

Characteristically, this hyperconnected chaperome network connects Hsp90 to Hsp70 via Hop and other immunophilins [74]. Additionally, chaperones with tetratricopeptide (TPR) domains augment the multimeric chaperone complex’s holdase activity by tightly bridging Hsp90/Hsp70 nucleation sites with Hsp40, Hsp110, and the sHsp pockets into a powerful, consolidated epichaperome capable of managing the proteostasis imbalance [6,75,76]. The resulting quaternary structure of the epichaperome offers multiple avenues for dynamic interactions and connectivity, permitting it to expand the functional capacity of the cancer cell in this overwhelming state.

In the prevailing chronic stress state, cancer cells adapt the epichaperome to overcome stoichiometric imbalances. There is an amplification of key chaperones including Hsp90, in addition to the epichaperome, to stabilize crucial proteins and form essential complexes despite component scarcity. To handle fluctuating stresses, they rewire protein interaction networks within the epichaperome, fine-tuning and swapping less critical components. Moreover, cancer cells, through mutations and epigenetic changes, alter protein interaction needs, improving protein folding and epichaperome homeostasis. Ultimately, cancer cells exploit the dynamic plasticity of the epichaperome condensates, which enables survival and proliferation even in the phase of fluctuating stoichiometric conditions.

Yet, this strength presents some vulnerabilities. Paradoxically, while the epichaperome remains robust in managing cellular stress, it becomes increasingly susceptible to inhibitors targeting Hsp90, particularly when coupled with defects in Hop and Hsp110 [76]. This vulnerability is evidenced by the differing interaction kinetics of Hsp90 with small molecules between normal and transformed cells that lack the *MYC* gene-reinforcing influence and cancer cells with the *MYC* reinforcement [6].

### 2.3. Molecular Regulators of Epichaperome Formation

Regarding the concept of the spontaneous assembly of biomolecules into functional units, epichaperome network formation equally follows this system in a thermodynamically favorable environment that delimits or accentuates their formation, interactions, and function [77]. The very environment dictates how they form and their cyclic client protein binding and release, a dynamic central to the epichaperome’s operation. Notable among these molecular regulators are post-translational modifications (PTMs) and transcriptional regulation [78,79], as well as co-chaperones and ATPase activity [80,81].

#### 2.3.1. Post-Translational Modification Regulates Epichaperome Activity

As covalent processes that modify specific amino acid sequences in proteins, PTMs modify the activity of proteins. For instance, acetylation affects the biological activity of Hsp90 [82]. The acetylation of Hsp90 at lysine residues 69 and 294 impairs its ATP-binding activity and interactions with co-chaperones and client proteins. Histone deacetylase 6 (HDAC6), an Hsp90 client protein, regulates Hsp90 activity by deacetylating it to maintain its functional conformation. Consequently, HDAC6 inhibition leads to increased Hsp90 acetylation, which affects its chaperoning functions [82]. We have also come to know that phosphorylating Hsp90 at Ser residues 226 and 255, which lie within its intrinsically disordered region (IDR), enhances its interaction with co-chaperones and drives its integration in the epichaperome as well as its epichaperome nucleation activity [83].

Hence, through PTMs, a wide range of sophisticated control is exerted on the epichaperome. These modifications fine-tune extensive epichaperome member protein structural and functional integrity. Likewise, PTMs also regulate the epichaperome’s avidity, affinity, and capabilities in a diverse array of functional outcomes.

#### 2.3.2. Transcriptional Regulations of Epichaperome Formation and Activity

Epichaperome induction and constitution are known to be regulated by heat shock factor 1 (*HSF1*) activation that transcribes Hsp genes by binding the heat shock element (HSE) domain [84]. The constitutive expression of HSF1 has been found in many tumors demonstrating its role in promoting epichaperome formation and control of proteotoxic pressure [85]. Characteristically, the upregulation of the MYC gene and its transcriptional factor activity promotes epichaperome formation [6].

Expanding on this research, Kourtis et al. established the link between *Hsf1* upregulation that promotes a T lymphoblastic-initiating cell population (MYC+LIC T-ALL cells) and the constitutive expression of *Hsf1* target genes, including Hsp proteins that form a functional epichaperome, an observation that was absent in genetically ablated *Hsf1* models resulting in a decrease in tumor size due to a decrease in the T lymphoblastic-initiating cell population (MYC-LIC T-ALL cells) [86]. Remarkably, NOTCH1 was noted to bind to the *HSF1* promoter region and many of its targets (48 out of 57 HSF1 targets), as confirmed by bioinformatic analyses. This indicates a potential role for NOTCH1 in oncogenic transcription and HSR pathways, a scenario that is absent in normal hematopoietic stem and progenitor cells. Considering the upregulation in HSR target genes, the authors proposed that NOTCH1 impacts epichaperome formation. NOTCH1 activation also predicted T cell acute lymphoblastic cell to chaperome-targeted therapy, while NOTCH1 pathway inhibition with NOTCH1 inhibitors influenced the levels of epichaperome formation, but it did not block its formation completely [86]. We have also come to understand that NOTCH1 binds within a broad super-enhancer region from the *MYC* transcription-initiating site and interacts with proximal sites in the *MYC* promoter [87] to activate a feed-forward-loop transcriptional network that promotes leukemic cell growth [88]. Nonetheless, the interaction of NOTCH1 in the locus of the *MYC* gene requires further investigation and proof regarding its direct connection with epichaperome formation in leukemias and other cancers. Moreover, patients with an unclassified myeloproliferative neoplasm harboring PML-SYK gene fusion show an upregulation of phospho-SYK, phospho-STAT5, phospho-ERK1/2, and phospho-S6 genes that drives a hyperactivated signal modulated by the epichaperome [89].

However, it has also been observed that Hsp90, together with Hsp70, synergistically binds and represses HSF1 activity [90,91]. In a study by Zou et al., a reduction in Hsp90 levels and not its co-chaperone and chaperonins increased HSF1 activation and transcriptional activity [91]. In response to cellular stress, Hsp90 dissociates from HSF1, allowing for Hsp transcription [91]. Therefore, a feedback loop regulates the transcriptional expression of Hsp90. In the cellular stress environment of cancer, this loop frees HSF1, allowing it to constitutively transcribe Hsp90, considering its indispensable role in the epichaperome.

#### 2.3.3. Co-Chaperones and ATPase Activity as Regulators of the Epichaperome

The integrity of the epichaperome is contingent upon a collaborative framework. This framework requires the co-chaperones and chaperonins in the network to maintain their position and collaborate with the epichaperome nucleation center: Hsp90 and Hsp70. Pivotal among the regulators of this nucleation promoter are Hsp70 and Hsp90 organizing protein (Hop), activator of Hsp90 ATPase (Aha1), C-terminus of the Hsc70-interacting protein (CHIP), and cell division cycle 37 (Cdc37) [6].

Numerous studies indicate that Hsp90 receives its client proteins from its obligatory collaboration with Hsp70, an interaction where Hop serves as a connecting link. Hop, with its two tetracopeptide repeat (TPR) domains, connects with Hsc70 via its N-terminal and with Hsp90 in the C-terminal through hydrophobic interactions in their conserved EEVD motifs in Hsp70 and Hsp90 [92].

Modeling the chaperoning of glucocorticoid receptor (GR), a client protein dependent on Hsp70/Hsp90 activity, Hop has been identified as a key modulator. Here, Hop regulates the cascades of Hsp70 inactivation, the formation of an inactive GR-Hsp90-Hop-Hsp70 loading complex that switches to an active GR-Hsp90-p23 maturation complex, and subsequently matured GR release. By potentiating Hsp90’s ATP hydrolysis activity and binding to all complex components, Hop promotes GR folding, remodeling, and maturation processes [93]. Hop is thus instrumental in chaperoning Hsp70/Hsp90 axis client proteins.

In the workings of Hsp90, it undergoes cycles of ATPase activity that change its conformation and facilitate its interactions with other co-chaperones, as exemplified above. In this axis lies the role of Aha1. By modulating different stoichiometric conformations of an Aha1/Hsp90 interaction, Mondol and colleagues demonstrated that two Aha1 (Aha1_2_) and Hsp90 dimers (Hsp90_2_) (Aha1_2_-Hsp90_2_) have the highest ATPase activity, followed by Aha1_1_-Hsp90_2_, and the least activity in dimeric Hsp90 [94]. Interestingly, the conformational state (open or closed structure) of Hsp90 did not change per stoichiometric interaction, revealing that the ATPase activity of Hsp90 does not promote a closed conformational structure. However, the number of Aha1 and Hsp90 interactions affects the unfolding force of Hsp90 [94]. Hsp90 unfolding is linked to its ability to promote local contraction in the unfolded chains of its client proteins to drive their global folding dimensions, and this is partly regulated by Aha1 [95].

Again, CHIP, an interacting partner of Hsc70, also interacts directly with Hsp90 heterocomplexes and abolishes its steroid-binding activity and transactivation potential on its client protein GR, leading to GR’s ubiquitin–proteasomal degradation. Thus, CHIP modulates the Hsc70/CHIP/Hsp90 triage decision to regulate proteostasis [96]. It is therefore important to have an increased Hop/CHIP ratio that promotes the loss of cancer cell regulation, a role that the epichaperome favors [97].

Favoring the pathogenesis of cancer cells, the epichaperome augments the folding and maturation of nascent and metastable kinases, which form a huge chunk of the epichaperome clientele. The sustained kinase activity promotes the aberrant signaling pathways, a hallmark of cancer [98]. The regulator of this activity is the Hsp90 co-chaperone, Cdc37. Cdc37 regulates this process by recruiting kinases to the epichaperome central axis via Hsp90-Cdc37 and a cyclin-dependent kinase 4 complex (Hsp90-Cdc37-Cdk4 complex). Hierarchically, Cdc37 recognizes client kinases via specific interactions in the Cdc37-Cdk4 complex and subsequently the whole Hsp90-Cdc37-Cdk4 complex, inducing conformational changes in the Hsp90-Cdc37-Cdk4 complex, which acts allosterically, suggesting a client-specific architectural dependence that ensures that different client proteins are chaperoned correctly and efficiently [99]. Consequently, the Hsp90-Cdc37 allosteric hotspots crucial for the Hsp90-Cdc37-Cdk4 complex conformational change [99] and related downstream PPIs arising from the refolding of metastable kinases have become a target for strategic therapy design [100]. Therefore, prospective research focused on unraveling the full complement of the dynamic interactomes and the associated signaling pathways of the epichaperome will be essential to understanding how these scaffolds could be exploited and regulated.

### 2.4. Signaling Pathways Following Epichaperome Constitution

#### 2.4.1. Canonical Signaling Pathways Regulated by Epichaperome

**Figure 1:**
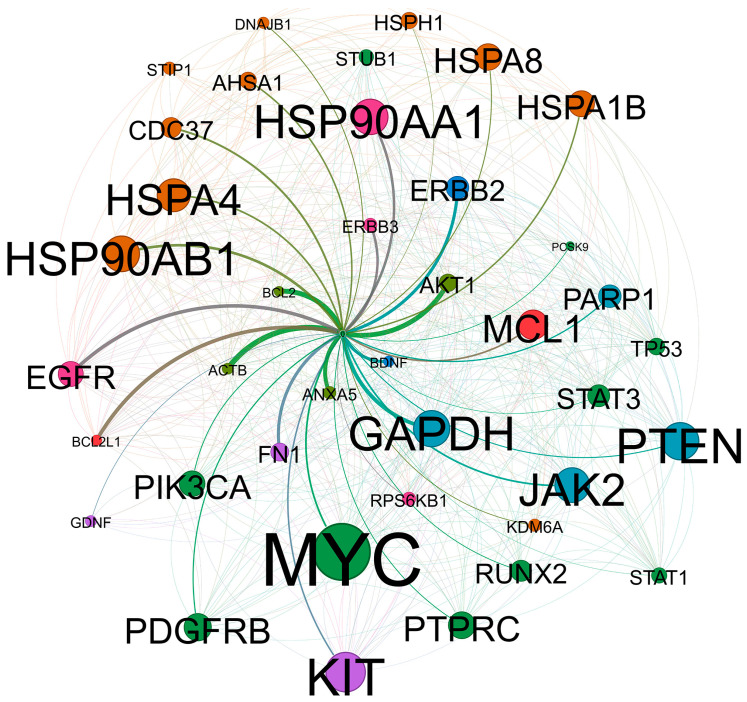
The epichaperome aberrant protein–protein and gene interactome showing some of the epichaperome’s oncogenic client proteins and genes it interacts with to prolong their cellular turnover, leading to dysregulated cell signaling as seen in cancers.

The assembly and subsequent activation of the epichaperome have been noted to initiate cascades of signaling events that impact cellular function, as summarized in Table 2. These assemblies not only disrupt established PPIs but also trigger new and aberrant PPIs that are essential for sustaining the pathological state of the cancer cell. Ultimately, these altered PPIs culminate in a gain-in-function PPI signal, a loss-of-function PPI signal, or a combination of both [101]. The ensuing interactome is often linked to aberrant MAPK signaling pathways, PI3K/Akt/mTOR signaling, NOTCH signaling, Wnt signaling, and Rb and TP53 signaling pathways [101]. A further analysis of biological functions following the inhibition of the epichaperome nucleation modulator Hsp90 revealed diverse proteome-wide changes. Remarkably, the induction of proteins involved in the 26S proteasome and, expectedly, the resultant downregulation of proteins involved in signal transduction pathways and nucleic acid metabolism (MPM-ALK, STIP1, IKKκ, MAK, and MCPH and a host of others can be found at https://www.picard.ch/Hsp90Int/index.php, accessed on 23 January 2025) occur due to their degradation [102]. In addition to the above-mentioned analysis, the pathway analysis revealed changes in JAK/STAT, TGFβ, PRAR, PDGFR, c-KIT, NF-κB, Anx, and KDM6 modulating apoptosis and integrin signaling components, as summarized in Figure 1.

The PI3K/Akt/mTOR signaling pathway, critical for metabolism, cell growth, and survival [103], is tightly regulated by the epichaperome via a plethora of mechanisms, such as the Hsp90 stabilization of the PI3K/Akt/mTOR signaling protein Akt that maintains its cellular levels, thereby influencing transformed cell survival [104].

The interplay between HSP70 and HSP90 in the nucleation, conformity, and stability of the epichaperome is crucial for tumor progression, making it a formidable therapeutic target. Investigating this in multiple myeloma, Chatterjee et al. reported that the PI3K/Akt/GSK3β signaling pathway regulates the expression of Hsp70/90 [105]. Specifically, Hsp72 and Hsp73 (Hsp70 family members) are overexpressed in multiple myeloma cells compared to normal plasma or B cells, and, as a result, therapeutically targeting these players induces apoptosis. The authors also noted that HSP90 functionality is dependent on HSF1, which is, in turn, controlled by the PI3K/Akt/GSK3β signaling pathway. Putting these together, the PI3K/Akt/GSK3β signaling pathway and the epichaperome system are dependently intertwined in cancer cells. In a causal loop system, JAK-STAT and PI3K/Akt signaling have been noted to promote tumor-enriched Hsp90 expression [106]. The importance of the epichaperome’s dysregulated control and interaction of this pathway is highlighted by the pathway’s frequent dysregulation in cancer. While disrupting this seems like a plausible strategy, the pathway is also critical for normal cell function. Therefore, therapeutic targets must focus on distinct mutant protein forms only found in tumor cells to spare normal cells from harmful side effects.

Nuclear factor-κB (NF-κB) signaling a crucial pathway that regulates inflammation and immunity [107]. Here, interactions between the epichaperome and IκB kinase (IKK) complex have been reported to modulate NF-κB activity and its downstream signaling [108]. The epichaperome’s impact on the NF-κB signaling pathways is context-dependent, leading to immunosuppression or, on the contrary, enhancing immunological responses [86,109].

Liang et al. have shown that heat shock protein beta-1 (HSPB1) overexpression in breast cancer is linked to EMT and IL-6-driven M2-type macrophage infiltration, and this promotes cancer progression and resistance to doxorubicin mediated by NF-κB signaling [110]. The authors reported that the ubiquitination-mediated degradation of lkβ-α, a key NF-κB transcription factor inhibitor that masks NF-κB’s nuclear translocation sequence (NLS) [111], enhances NF-κB nuclear translocation that modulates the downstream malignant cascades. Moreover, Liu et al. found that an increased Hsp90 expression in gastric cancer cells correlated with elevated EMT-related markers—specifically N-cadherin, E-cadherin, vimentin, and Snail—and a greater proportion of CD90^+^ proliferative and CD44^+^ metastatic stem cells when compared to cells with blocked Hsp90 expression [112]. Conversely, Hance et al. showed that extracellular Hsp90 (eHsp90) modulates a significant shift towards an EMT phenotype in androgen-repressed prostate cancer cells (ARCaPE cells) through the eHsp90-LRP1 modulation of FAK and ERK signaling [113]. eHsp90 suppressed E-cadherin expression while simultaneously boosting N-cadherin and Twist, accompanied by a morphological transition to a mesenchymal-like cell shape that showed a more aggressive and invasive behavior. Blocking eHsp90 reversed this phenotypic behavior. The EMT modulation regulated by Hsp90 (intracellularly) is orchestrated through the STAT3 signaling pathway, where Hsp90 stabilizes STAT3 and promotes its binding to the *TWIST* promoter, upregulating Twist transcription [114].

In response to stress and inflammation, heat shock proteins such as Hsp70 and Hsp60 have been shown to modulate the MAPK pathways (ERK, JNK, and p38) via multiple mechanisms. This includes direct and indirect interactions affecting MAPK cascade components, upstream regulators, and ultimately, the activation, localization, substrate specificity, and MAPK-pathway-related genes [115].

The short mucin-like cell surface protein CD24 has been reported to promote colorectal cancer by driving pro-angiogenic signals. Investigating this, Wang et al. found CD24 upregulation activates STAT3 phosphorylation, which promotes VEGF production and stimulates angiogenesis via an HSP90-dependent STAT3/VEGF signaling pathway [116]. Critically, targeting the CD24-Hsp90 interaction emerges as a potential therapeutic approach for colorectal cancers. For clarity, prospective studies should decipher the exact CD24-Hsp90 interaction pathway and identify biomarkers linking CD24 expression to patient outcomes. Equally, Hsp90 inhibition has been linked to the downregulation of STAT3 and TWIST1 transcription pathways, thereby inhibiting tumor progression, metastasis, and chemoresistance in diverse tumors [114].

Besides these pathways, Hsp90 is known to modulate the Bcr-Abl pathway, whose mutation leads to various forms of leukemia [117]. Also, it modulates the Wnt/β-catenin and EGFR and Her-2 pathways, all of which are Hsp90 client proteins [117]. Hence, the knockdown or inhibition of Hsp90 directly or indirectly affects these pathways.

Overall, the epichaperome network potently chaperones mutant signaling pathway proteins to accentuate their functional cycle to “hijack” and aberrantly regulate cellular functions, which promotes the phenotypic picture seen in cancers. It thus supposes that the structure and function of the epichaperome might vary among malignancies and even within the cellular heterogeneity, explaining intratumoral and intertumoral differences in aggressiveness, pathogenesis, and metastasis.

**Table 2:** Epichaperome and related intracellular signaling pathways.

Signaling Pathway	Molecular Chaperone	Function	Reference
PI3K/Akt/mTOR	Hsp90	Stabilizes the PI3K/Akt/mTOR pathway signaling protein Akt, maintaining its cellular levels, thereby influencing cancer cell survival.	[6,104]
		Hsp90 inhibition leads to PI3K/Akt and EFGR pathway shutdown.	[6,117]
	Hsp70/90	PI3K/Akt/GSK3β signaling crosstalks with Hsp70/Hsp90 expression by inducing HSF1 expression in multiple myeloma.	[105]
NF-κB	Epichaperome complex	Interacts with the NF-κB signaling pathway in a context-dependent manner, causing immune-limiting or -enhancing responses to drive tumor growth.	[86,109]
	HSPB1	Modulates EMT and IL-6-driven M2-type macrophage infiltration that promotes cancer progression and resistance to doxorubicin by modulating NF-κB signaling.	[110]
MAPK	Hsp70	Interacts with MAPK to modulate the processes of hypoxia, inflammation, and apoptosis.	[115]
	Hsp60	Stimulates vascular smooth muscle cell migration by interacting with MAPK.	[115]
STAT3/VEGF	HSP90	Maintains the cell surface membrane expression levels of CD24 by stabilizing it to promote sustained STAT3 phosphorylation, which promotes VEGF production and stimulates angiogenesis in colorectal cancer. Hsp90 inhibition, in addition to STAT3, downregulates TWIST1 gene transcription that slows tumor growth and promotes chemoresistance.	[114,116]
JAK/STAT, TGFβ, PRAR, integrin	Hsp90	Chaperone MPM-ALK, STIP1, IKKκ, MAK, MCPH, and other related proteins to maintain the JAK/STAT, TGFβ, PRAR, and integrin signaling pathways.	[102]
Bcr-Abl/Wnt/β catenin/EGFR & Her-2	Hsp90	Client proteins of Hsp90, the inhibition of which results in the downregulation of these pathways.	[117]

#### 2.4.2. Epichaperome and Modulation of Cancer Stem Cell Signaling

A well-established observation in cancer is the formation of a subset of cells, the cancer stem cells (CSCs), that are driven by some positive pressures [118]. CSCs are forged by persistent stress and selective pressures—including radiochemotherapy [119], hypoxia [120], and the dynamic extracellular matrix [121]—that rewire cancer cell chromosomal landscapes and signaling pathways and induce the epichaperome formation, driving the emergence of CSCs [83]. A hallmark of this emergent phenotype is an inherent capacity of self-renewal, governed by the transcription factors Nanog [122,123,124], Sox2 [124], Oct4 [124,125], MYC [126], and STAT3 [123,127]. These regulators control key signaling pathways—Wnt/β-catenin, NF-κB, Notch, Hedgehog, YAP and TAZ, TGF-β/SMAD, PI3K/Akt, VEGF/HIF-1α, MAPK, JAK/STAT, and Hippo—essential for driving and sustaining CSC stemness in both solid and hematogenous cancers [118,128,129].

To illustrate this complex interplay, Sato et al. researched IL-6-induced STAT3 signaling, a crucial regulator of human hepatoma cell stemness, a feature that was diminished upon the inhibition of Hsp90 with geldanamycin [130]. They established STAT3 as an Hsp90 client protein, binding STAT3 via its N-terminal region. Indeed, the role of Hsp90 in maintaining stemness in human embryonic kidney carcinoma 293T cells is evident, a feature that is Hsp90-dependent and lost when Hsp90 is inhibited, while its upregulation restores this phenotype [130]. Further, Bradley et al. showed that Hsp90 modulates Oct4 mRNA levels while chaperoning the stem cell markers Oct4 and Nanog to regulate pluripotency and facilitate cancer cell plasticity [131]. As you would expect, Hsp90 inhibition by 17-N-allylamino-17-demethoxygeldanamycin or miRNA activates mesodermal lineage protein markers to promote the mesodermal differentiation of embryonic stem cells. Remarkably, Liu and colleagues observed Hsp90 overexpression in gastric cancer cell lines that elevated stemness (CD44, Nanog, Sox2, and Oct4) and EMT markers, a defining characteristic that was absent in the Hsp90 knockdown cells [112]. The downstream intracellular signaling pathways that ensue following these intracellular changes control the cancer cell behavior.

In acute myelogenous leukemia (AML), the epichaperome formation stabilizes and maintains the activity of oncogenic kinases and the mutant p53 transcriptional factor, consequently promoting PI3K/Akt/mTOR, MAPK/ERK, and STAT3, c-MYC, as well as HIF-1α signaling pathways [132]. This sustained signaling drives and maintains the stemness of AML stem/progenitor cells and TP53-/kinase mutant AML cells, influencing tumor growth and survival [132]. Notably, epichaperome inhibition enhances the efficacy of venetoclax, overcoming venetoclax-resistant TP53-/kinase mutant AML cell clones. Similarly, in triple-negative breast cancer cells (TNBCs), targeting Hsp90 with celastrol and triptolide disrupts the TNBCs stemness by inhibiting Notch signaling [133], further highlighting the stemness-maintaining role of the epichaperome.

Studies by Vu and Kharas’s groups demonstrated that the depletion of synaptotagmin-binding cytoplasmic RNA-interacting protein (SYNCRIP), an mRNA-binding protein that regulates gene expression, interrupts protein homeostasis in hematopoietic stem cells (HSCs), suppressing Rho GTPase effectors and signaling [134]. Intriguingly, pathway enrichment analysis revealed that SYNCRIP depletion triggers a compensatory HSF1/Hsp70/Hsp90 response, ultimately leading to epichaperome formation. Epichaperome formation, in turn, stabilizes Rho and Rho-associated effectors and signaling proteins to sustain Rho GTPase signaling, restoring crucial HSC activities such as proliferation, polarity, migration, cytoskeletal organization, and adhesion [134], drawing a linkage between mRNA processing, the epichaperome, and stem cell function.

The influence of the epichaperome constituents in regulating CSC stemness extends beyond the cell’s intracellular domain as extracellular Hsp90 (eHsp90) plays a pivotal role. Nolan and colleagues, investigating the role of eHsp90 in the dynamics of CSC stemness, reported that Hsp90 promotes tumor cell invasion and metastasis by upregulating cancer cell stem-like gene targets and EMT markers and enhancing prostasphere formation [135]. Furthermore, it should also be noted that homologs of the epichaperome core actors also contribute to the regulation of tumor stemness. TRAP1, for instance, promotes stemness in colorectal carcinoma through Wnt/β-catenin activation [136]. In the same vein, GRP78 maintains breast cancer cell stemness via the β-catenin/ABCG2 signaling pathway [137,138], with extracellular GRP78 also implicated in tumor cell stemness [139,140], re-echoing the possibility of a multicompartment intracellular epichaperome network that connects extracellularly, further shaping tumor cell behavior.

In summary, the epichaperome’s role in tumor cell survival and growth is strikingly similar to its role in stem cells, enhancing the apoptotic threshold and modulating the CSC’s metabolic state, cancer cell plasticity, and drug resistance. However, whether epichaperome formation is a universal characteristic of all tumor and cancer stem cells remains a crucial, yet unproven, assumption. Could the scenario that some but not all tumor cells, depending on an upregulated MYC gene expression, form epichaperomes pan out in the CSC population? However, given that CSCs characteristically form epichaperomes, another critical question arises: could conditions that induce epichaperome formation in tumors also drive the dedifferentiation of tumors cells towards a stem-like state? What is the threshold of these dynamics in context? These transition dynamics require attention in therapeutic strategies to effectively eradicate these radiochemotherapy-resistant cell populations. Yet, unlocking this remarkable possibility hinges upon a deep introspection into epichaperome biology.

#### 2.4.3. Epichaperome Regulation of Autophagy Signaling

Autophagy is closely linked to proteostasis and crosstalk with the epichaperome [141], with several chaperones like Hsp70 and Hsp90 to regulate autophagy initiation and cargo selection via the mTOR signaling pathway [142]. The autophagy’s complex machinery involves the ULK1/2 kinase core complex, the autophagy-specific class III PI3K complex, the ATG9A trafficking system, ATG12, and the LC3 ubiquitin-like conjugation system [143]. The role of autophagy in cancer is context-dependent, with an initial tumor suppressive role that hinders early tumor growth [144,145]. Paradoxically, autophagy can shift to a tumor-promoting conformation in established cancers, supporting tumor cell survival, growth, metastasis, and inflammation [143,146].

In a recent study, using 3-methyladenine (3-MA)- and lonidamine (LND)-co-encapsulated liposomes designed to inhibit autophagy and hexokinase, Zhao et al. shut down autophagy and HSPs, thereby significantly weakening tumor cell thermotolerance to radiofrequency ablation and consequently tumor suppression [146]. In a comparable study, Wang et al. used a bone marrow mesenchymal stem cell (BMSC) model to explore the interplay between Hsps and autophagy pathways. They discovered that heat-shocked BMSCs (HS-BMSCs) treated with cisplatin showed reduced apoptosis compared to their non-heat-shocked BMSCs [147]. Interestingly, HS-BMSCs also exhibited a decrease in autophagy markers (Beclin1, the LC3BII/LC3BI ratio, and autophagosomes) along with an increase in Hsp90 and Hsp70 expression, suggesting that heightened Hsp expression can suppress autophagy and control proteostasis. This indicates a compensatory mechanism between these pathways, suggesting that effective tumor treatment may necessitate suppressing both autophagy and the epichaperome.

#### 2.4.4. Epichaperome and Nuclear Signaling Dynamics

The regulation of nuclear signaling is a well-coordinated endeavor where the regulatory role of the epichaperome extends. For instance, in cell growth and gene expression, Hsp90 forms a complex with its nuclear co-chaperone R2TP, the Hsp90/R2TP complex, to regulate the assembly of multi-protein complexes crucial for the biogenesis of various ribonucleoproteins (RNPs), including L7AeRNPs (C/D and H/ACA SnoRNA), telomerase, the spliceosome snRNPs, U4, and selenoprotein mRNPs [148]. The Hsp90/R2TP complex also interacts with RNA Polymerase II (RNAP II), suggesting a role in its assembly [149]. A recent study by Maurizy et al. revealed that the HSP90/R2TP co-chaperone complex is essential in intestinal cell homeostasis [150], with Rpap3 deficiency disrupting RNAP II nuclear transport that downregulates mTOR, ATR (DNA damage response), and ATM (DNA strand break response) signaling. Paradoxically, Rpap3 overexpression correlates with colon cancer progression and poor prognosis [150]. Mechanistically, the HSP90/R2TP complex appears to influence cell cycle progression via cyclin D1 and E2F, hinting at a potential linkage and possible regulatory role of the HSP90/R2TP complex to the WNT signaling pathway. Hence, future research should focus on identifying the HSP90/R2TP complex-specific client proteins regulated within the intestinal epithelial cell (IEC) milieu. Equally important is the investigation of intestinal cell subtype-specific effects and exploring therapeutic strategies and targets.

Furthermore, Hsp90 interactions with Lamin-A could potentially regulate DNA damage repair and chemoresistance in ovarian cancer. Consistent with this, Wang et al. reported that Hsp90 interacts with the nuclear pore protein, Lamin-A, via its 1-430 domain to mediate Hsp90 nucleocytoplasmic transport [151]. Inhibiting Hsp90 with cisplatin reduced DNA double-strand repair, stalling cell proliferation. Combining cisplatin with 17-N-amallylamino-17-demethoxygyeldanamycin suppressed tumor growth and metastasis using a mouse xenograft model [151]. All in all, further research into the mechanisms of HSP nuclear transport, the specific roles in DNA repair pathways affected, and synergistic effects of combined small molecules and chemoradiotherapy could be a promising avenue that would inform clinical applications for other cancer treatments and other cancers at large.

#### 2.4.5. Epichaperome Regulation of Mitochondrial and Metabolic Signaling Pathways

Like most pathways, metabolic signaling is also modulated by protein–protein interactions. Hence, the role of proteostasis cannot be overemphasized in the dynamics of the tumor cell bioenergetics. A hallmark of the cancer metabolic picture is the “Warburg Effect”, where aerobic glycolysis becomes the preferred energy generation mechanism [152,153,154].

Here, Hsp90 and Hsp70 are central to the shift to cancer metabolism. By modulating the mitochondria voltage-dependent anion channels (VDACs), Hsp90 directly influences this metabolic shift [155]. Moreover, Hsp90 interacts with oncogenic signaling pathways like cMYC [156], HIF-1α [157], and PI3K/Akt [158] pathways to further drive the transition from oxidative phosphorylation (in normal cells) to aerobic glycolysis in cancer cells. For instance, Hsp90 interacts with and stabilizes the structural and functional integrity of cMYC [156] to regulate NAMPT gene expression and a host of other mitochondrial genes that are involved in NAD+ synthesis to alter cancer cell metabolism [159,160]. Likewise, Hsp70 overexpression can inhibit oxidative phosphorylation and compensate for the reduced ATP production through enhanced glycolytic activity. Probing this, Wang et al. demonstrated that by repressing NADH dehydrogenase and ubiquinol-cytochrome-c reductase while enhancing phosphofructokinase and lactate dehydrogenase activity, Hsp70 compensated the ATP balance while downregulating the oxidative phosphorylation pathway [161]. In response to cold shock, Hsp70 overexpression increased metabolic kinase activity via constitutive Akt pathway upregulation to increase glycolysis and glycogen synthesis [162].

In a seminal study that employed multiomics and systems biology approaches, Poverennaya et al. characterized the functional annotation of TOMM34, a mitochondrial membrane trafficking protein [163]. In the dynamics of mitochondrial protein trafficking, TOMM34 forms a complex with Hsp70/90 (the Hsp70-TOMM34-Hsp90 complex) [164] that is upregulated in many cancers [162,165,166,167,168]. A comprehensive analysis of the multiomics data revealed that TOMM34 interacts with deregulated cancer-associated pathways such as NOTCH, MAPK, and STAT3 signaling pathways. While the precise role of Hsp90/Hsp70 in this complex is not fully understood, the translocation capabilities of TOMM34 enhance NOTCH, MAPK, and STAT3 ligand translocation into the mitochondria, whereas the ATPase activity of Hsp90/Hsp70 in the complex enhances the constitutive activation and activity of NOTCH, MAPK, and STAT3 signaling. This translocation then promotes tumor cell survival (NOTCH), regulates metabolism (MAPK), and enhances ATP production (STAT3). But further research into the precise regulatory capabilities by elucidating the PPIs of the Hsp70-TOMM34-Hsp90 complex with these signaling pathway constituent partners of proteins will provide an insight into the cancer cell mitochondrial biology.

A careful study of cancer biology has revealed that tumor cells exhibit high levels of TRAP1 within their mitochondria to maintain protein homeostasis [169]. Research by the Altieri group reported reduced ATP production and overall tumor cell energy metabolism characterized by reduced glucose utilization and lactate production following TRAP1 knockdown with Gamitrinib [170]. Hence, the stabilization of the bioenergetic sensing and modulation pathways of the tumor cell may rely heavily on chaperones through an mTOR-dependent pathway [171]. Further studies looking at TRAP1, Hsp70, and Hsp90 and their broader interaction maps within the mitochondrial network will be very informative. Prospective works should also explore the effects of targeting these chaperones on the hallmarks of cancer.

## 3. Liquid–Liquid Phase Separation in Epichaperome

Efforts to describe the dynamics of molecular events occurring in the cell and in its different compartments, though intensely researched over the past few decades, are mainly inconclusive, with many unanswered questions. A plausible explanation for the transient separation and reversible merger of macromolecular droplets, called condensates, is that the phase transition phenomenon presents an empirical concept for understanding molecular interactions.

The formation of epichaperome condensates, like most macromolecular condensates, depends on weak, multivalent interactions between client and scaffold proteins [172]. These interactions, driven by electrostatic, hydrogen, and hydrophobic forces, reversibly influence the strength (avidity) of PPIs to promote the liquid–liquid phase separation (LLPS). Intrinsically disordered regions (IDRs), particularly in Hsp70 and Hsp90, are rich in interaction motifs, such as the HEAT motifs and TPR domains that facilitate their conformational switching for transient multivalent interactions [173,174,175]. Additional to the IDRs are prion-like low-complexity domains, which are equally vital for the phase separation process. For instance, the IDRs of the Hsp90, as shown in the Ser226 and Ser255 residues, modulate epichaperome formation [83]. More so, prion-like low-complexity domains like TDP-43 can act as a dynamic switch to influence epichaperome nucleation [176]. In short, IDRs and prion-like low-complexity domains are molecular features that could modulate the molecular hub and RNA processing capabilities of the epichaperome, making them important for research into its phase separation patterns.

The nature of these structural and valence properties of interactomes, influenced by PTM processes, ionic concentrations, and pH levels, dictates the attractive and repulsive forces that drive the rapid formation and dissolution of cellular condensates [177]. This precise assembly/disassembly process, which occurs in precise spatiotemporal patterns in response to cellular signals, is critical for processes such as signal transduction.

Indeed, many diseases, including cancer, develop from aberrant signaling processes. Cell signaling pathways utilize liquid–liquid phase separation (LLPS) to enhance precision and specificity. But the disruption of the rather well-organized LLPS process in intracellular signaling has been linked to cancer development and progression [178,179]. Aiding this aberrant process are the epichaperome network mediators, HSP90 and HSP70. Testing this hypothesis, the Jin Zhang group demonstrated in atypical liver cancer that a fusion oncoprotein, PKAcat, interacts with DnaJ chaperones like DnaJB1 (DnaJB1-PKAcat) to prevent myristylation (a lipid PTM process that signals downstream apoptosis), a key PTM process that normally leads to apoptosis [180]. Of note, this interaction also enables PKAcat to bind Hsp70, which stabilizes its structural and functional conformation. As a result, there are suppressed cellular cAMP levels, leading to an abnormal LLPS of regulatory subunit 1α (R1α), ultimately leading to uncontrolled cell growth.

Furthermore, the role of Hsp90 in stress granule (SG) dynamics appears to be relevant to cancer, for cancer cells show Hsp90 upregulation and dysregulated mTOR signaling. In the SG formation, a dynamic ribonucleoprotein protein assembly, molecular condensates that sequester stalled mRNAs, the mTORC1 scaffolding protein raptor is sequestered [181]; however, the dual-specific tyrosine-phosphorylation-regulated kinase 3 (DYRK3) promotes SG dissolution after stress to restore mTORC1 signaling [182]. The sequestration of the mTORC1 scaffolding protein in the SG typically protects cells from apoptosis, but in cancer, this process is distorted. The enhanced Hsp90 presence in tumor cells promotes DYRK3 LLPS, which then increases the activity of p62 by phosphorylating it at the Ser-207 and Thr-269 residues, to in turn phosphorylate the mTOR1 protein via TRAF6 phosphorylation as an intermediary [183]. The linkage of their direct causal role in the mTOR1 signaling pathway checks out when DYRK3 and/or p62 are inhibited, blocking mTOR1 activity. In a related study, Serena Carra and Simon Alberti’s groups reported SG dissolution and DYRK3 stabilization and quality control to be HSP90-dependent, such that the HSP90/Cdc37/HSP70 complex inhibition delays the reversible SG phase separation and disassembly process [179,184].

Summing up, it is our view that these LLPS dynamics play out in the epichaperome in tumors, with Hsp90 and Hsp70 as an “ignition plug” that stabilizes oncoproteins. The epichaperome maintains the phase boundaries of oncoproteins and provides them with a thermodynamically favorable heat capacity and Gibbs free energy [77], which aids the swiftness of the LLPS process to maintain the transformed cellular state. Hence, modulating the interactions of the epichaperome network or altering the expression levels of key components through specific PTMs could disrupt interactions and the thermodynamic environment they create, and this could be a promising avenue. Therefore, the determination of the epichaperome condensate architectural structures using advanced single-molecule imaging techniques will provide empirical insights into the functional organization of the epichaperome.

## 4. Techniques for Identification of Epichaperome Constituents and Network

### 4.1. Method for Isolating Epichaperome

Cellular response to molecular cues and stressors is executed via protein translation and their dynamic spatiotemporal networks, which are coded in the primary source of information (the genome) [73,185]. Intriguingly, the genome itself and its products, the proteome, interact in a causal loop system in what is broadly termed the interactome [186]. As such, the proteome at any given time presents an introspective map of the cell’s state and primary information source. In this background, isolating proteins or the proteome in a context-specific manner could provide an invaluable map representative of the functional impact of the genome explained by protein–protein interaction networks [40].

A crucial step prior to the identification and functional characterization of intracellular protein complexes is context-specific isolation. Moreover, the range of methods, including mechanical (homogenization, sonication, ultracentrifugation, and freeze-thawing) and non-mechanical approaches (chaotropic and enzymatic), is limited due to protein denaturation and the loss of activity [187]. Therefore, an optimal approach is necessary to fully appreciate the functional changes in native biological systems.

Our current abilities transcend developing chemical toolboxes, such as chemical probes, for research; these tools can also be engineered for strategic therapeutic applications. In this respect, the use of chemical probes as baits (such as YK5-B, YK6 beads, fluorescently labeled PU-H71, and PU-H71 beads [6]) is gaining attention considering the limitless possibilities of employing these in a context-specific manner, isolating specific epichaperome interactors together with their intact networks for omics studies (epichaperomics) [188] and applying the same method to strategically target epichaperome constituents [189]. Competitively, these chemical probes mitigate the exogenous introduction of tagged purified proteins, which come with their own inherent limitations [190].

**Figure 2:**
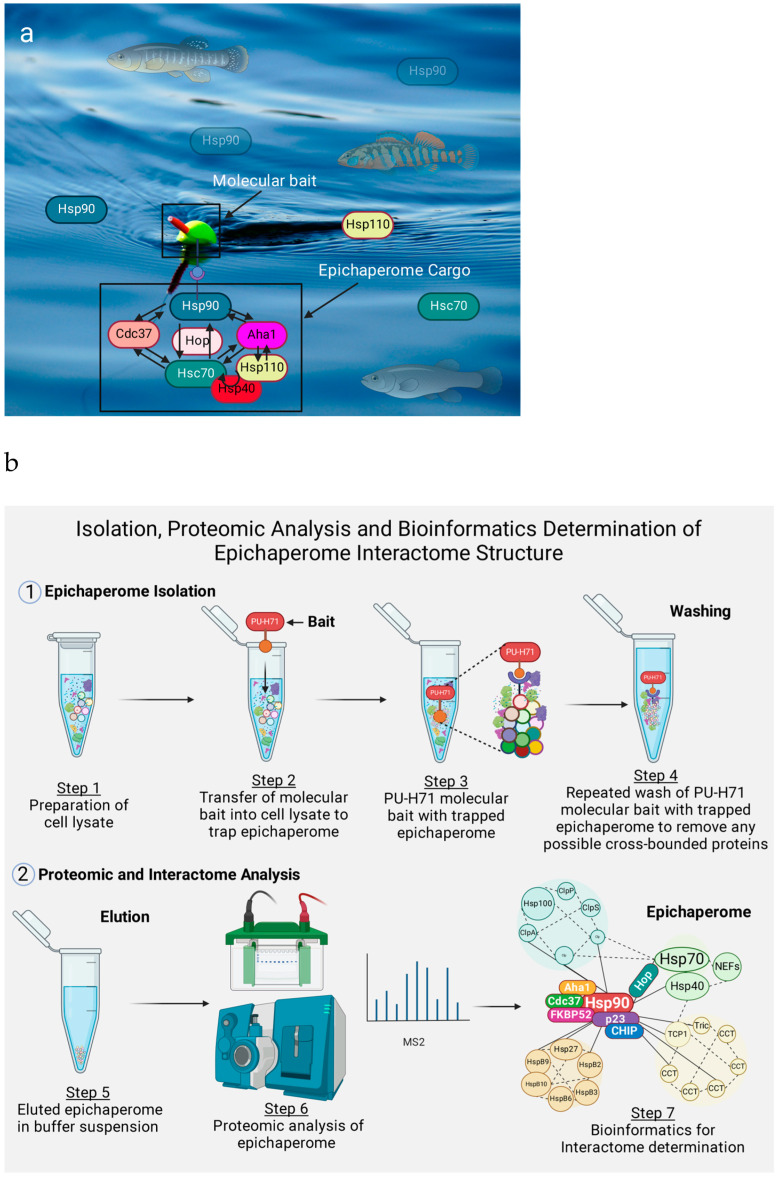
A schematic workflow of proteome capture through to bioinformatic analysis of the isolate. (**a**) A hook, line, and sinker representation of molecular bait that has selectively captured a fish of the epichaperome cargo. (**b**) Through the respective stages, the capture is washed a couple of times to remove any possible cross-bound proteins, after which the cargo is eluted with an elution buffer, then the proteomic analysis is conducted, and finally, the bioinformatic analysis is carried out to determine the interactome.

In a manner comparable to the hook, line, and sinker for fishing (as illustrated in Figure 2), molecular baits operate by selectively binding to their target proteins. A process known as affinity capture enables the engineering of an epitope (molecular bait) for a specific high-affinity interaction with its paratope (“prey”) (and vice versa). By attaching biotin (biotinylated YK5-B probes), molecular baits covalently tag and capture endogenous proteins together with their interactors within a 10–20 nm range [188]. Remarkably, the epichaperome baits are engineered based on molecular chaperones. These probes bind there like chaperones in the epichaperome network and are reorganized into the epichaperome, allowing the isolation of the entire epichaperome network together with its functional connectors, allowing for the capture of the functional range of the cell and for pulling down the epichaperome machinery [188]. Following the capture, baits are washed with buffers to remove any “promiscuous” proteins that can be competitively washed away because they bind weakly. The ensuing isolate is considered the captured protein or proteome, initiating the proteomics pipeline.

While molecular baits have revolutionized the approach to studying the epichaperome, they are not without limitations. The inherent limitations of affinity-based approaches, particularly when studying the dynamic epichaperome, present significant hurdles. For instance, the steric bulk of biotinylated probes can impede accurate capture, while the transient nature of the epichaperome interactions means that crucial interactors may be lost during the protein extraction and homogenate pull-down procedures. Moreover, homogenate preparations can induce misfolded proteins, leading to the spurious capture of non-specific interactors and a surge in background noise that compromises the accuracy of proteome identification [191]. Adding to these challenges, the use of PU-H71 and YK-6 beads is hampered by non-specific binding, difficulty in the release of captured proteins, and the laborious protocols that restrict throughput applications. Moreover, fluorescently labeled baits are likely to photobleach or suffer the effects of autofluorescence. In this regard, suggestions for lipid-engineered or viral receptor-designed baits that can orchestrate cell membrane crossing and membrane-specific homologs of Hsp90 and Hsp70 could abrogate the limitation of lysing cells, hence paving the way for the spatial and temporal mapping of the epichaperome network in intact cells.

In the same vein, current protocols are mainly based on cell lysates. For this reason, mapping out the spatiotemporal network of the epichaperome is a challenge. Additionally, baits could be engineered to recruit protein-tagging enzymes in a proximity-dependent manner, enabling the site-specific labeling of epichaperome protein interactors in specific membrane compartments as another approach to surmounting the limitation of spatiotemporal mapping. Furthermore, multiple protein tags for labeling specific epichaperome subnetworks while inducing physiological perturbations could help study how the epichaperome responds to differing physiological cues and the dynamic integration thereof. Lastly, the development of mathematical models incorporating spatiotemporal data could provide unprecedented insights into the mysteries of the epichaperome’s workings.

### 4.2. Identification of Epichaperome Constituents and Network

The discovery of what has now become known as molecular chaperones initially originated from heat shock proteins stemming from the serendipity of Ritossa’s Drosophila melanogaster experiments in the early 1960s. Early research into molecular chaperones relied heavily on biochemical and cell biology techniques. The proteins were induced using cellular stress, separated via SDS-PAGE electrophoresis to distinguish their unique identities, and then purified with classic column chromatography. These methods fueled the discovery of the first molecular chaperones, Hsp70, Hsp60 (GroEl in bacteria), Hsp90, small Hsp (sHsp), and many others, as we have come to know today.

While classic protein study methods like SDS-PAGE electrophoresis and chromatography remain relevant, the need for understanding molecular chaperones and their intricate interactions at a systems level requires advanced approaches. At this scale, cell-based models like the yeast two-hybrid (Y2H) screen with specific chaperone knockdown strains for functional gene analysis coupled with computational biology techniques, mass spectrometry methods like MALDI-TOF/LC-MS/MS, and Northern blotting have become essential for mapping out chaperone PPI frameworks [192]. These techniques are critical for studying cancer PPI pipelines, where the identification of the epichaperome [6] has revealed the complex PPI pipelines in cancer and normal cells. The characterization of these complex workings of the protein dynamics requires a comparative analysis of protein abundance, protein expression changes, chaperone–client protein interactions, and their associated PTMs. However, the transient dynamic nature of proteins presents another barrier that ought to be addressed. Approaches like cross-linked mass spectrometry (XL-MS) have enabled the study of transient PPIs, facilitating the discovery of new molecular chaperones and the characterization of their functions [193,194].

In addition, cytometry-based assays present another layer to evaluating and monitoring epichaperome abundance at the single-cell level [89]. Quantitative proteomics and affinity purification–mass spectrometry approaches, such as label-free quantification (LFQ), SILAC, the use of tagged chaperone as “baits” to capture and identify client proteins, synthetic genetic array technology (SGA), and advanced bioinformatic tools, have revolutionized network analysis by integrating proteomic and functional genetic data to map out global genetic and protein interaction networks [195].

However, despite these advancements, some limitations remain to be surmounted [196]. The Y2H model is limited in its capacity to detect PPIs, study PTMs, and assess the conformational status of proteins [196]. Mass spectrometry techniques like MALDI-TOF/LC-MS/MS are challenged by the transient, dynamic nature of PPIs and the inherent background noise, making the comprehensive global characterization of some PPIs a challenge [197]. Consequently, some PPIs may go unidentified or unnoticed. Moreover, the condensate bulk proteomics approach employed in most studies is likely to miss out on changes in cellular heterogeneity and localized cellular processes in different compartments seen in the tumors cell. In the same vein, SILAC-based IP-MS relies on complete metabolic labeling, which itself can affect protein interactions and cellular processes [198]. Hence, there is a need for high-throughput systems that augment the detection and quantification of the spatiotemporal resolution of PPIs and the redress of the limitations noted above.

To this end, we propose the incorporation of spatial single-cell multiomics studies in cell-based models to gain a high-resolution capture across multiple layers of molecular information that furthers an integrated understanding of cell heterogeneity and regulatory mechanisms. There is also a need to develop and incorporate high-resolution single-molecular proteomics using techniques like imaging mass cytometry and dynamic SILAC that would allow time-resolved spatiotemporal chaperone expression and interaction mapping within the context of the tumor. Quantitative proteomics methods, such as DIA and PRM, can be used for the high-throughput screening of chaperone inhibitors and combination therapies. Without delay, high-specificity chemical probes that modify chaperones and their client proteins will help elucidate their activity, dynamics, and abundance in situ. Finally, integrating multiomics (proteomics, transcriptomics, and phosphoproteomics) data with artificial intelligence/machine learning will uncover novel patterns and biomarkers for patient stratification and drug–target combinations. It is our view that a comprehensive approach will pave the way for an in-depth understanding of epichaperome biology and drug targets.

## 5. Implications of the Epichaperome for Cancer Therapy and Therapeutic Design

Tumors, akin to a complex ecosystem, harbor diverse cell populations that are shaped by the forces of the cancer stem cell (CSC) [118] and clonal evolution cell theories [199] or a combination of both amidst other theories. This suggests that spontaneous genetic and epigenetic events within tumor cells could give rise to diverse, selectively favored clones to drive tumor growth.

The selectivity and heterogeneous diversity in the tumor cell populations are supported by diverse clonal fates that emerge in the tumor microenvironment upon the treatment of homogeneous cancer cells [200,201]. Current targeted treatments of the epichaperome, like the PU-H71 small molecule, are based on the principle of selectively targeting the nucleation site, specifically Hsp90, of epichaperome-expressing cancer cells. This small molecule has shown promising results [202,203,204]. Uniquely, the thermodynamics of the PU-H71-Hsp90 interaction show a predilection for active epichaperomes, dissociating rapidly compared to their unbound epichaperome counterparts, which allows for the preferential targeting of active epichaperomes [189]. This specificity has led to success in treating cancers like PML-SYK-fusion acute myeloid leukemia. After 16 rounds of PU-H71 administration, the Guzman group reported that the patient achieved enduring complete remission, which is quite remarkable [89]. Nonetheless, this feat is far from universal. Comparative studies assessing current therapeutics that target epichaperome have become imperative. Further investigations into their efficacy and limitations are urgently needed to optimize cancer treatment. Only then will we be able to create epichaperome-directed treatments that are effective.

**Figure 3:**
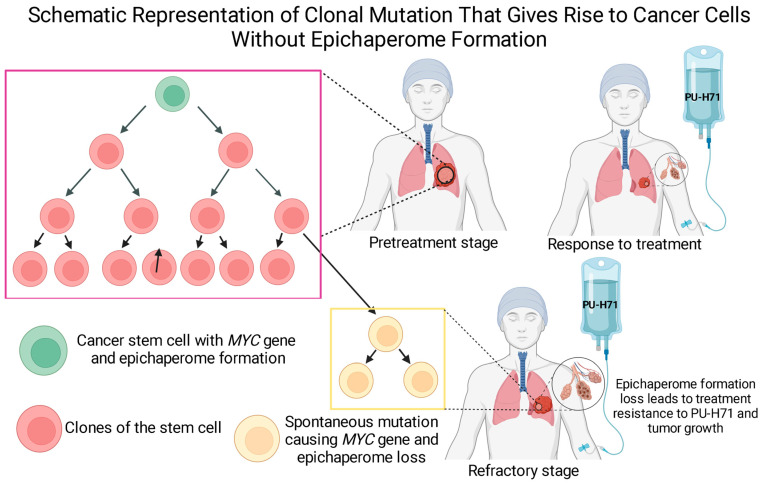
A scheme of the refractory treatment stage that arises in the course of treatment with PU-H71 due to a possible spontaneous mutation in a cancer cell clone that leads to the loss of the MYC gene and, consequently, the lack of epichaperome formation, making these clones resistant to PU-H71.

This is because the tumor microenvironment is a dynamic battlefield. Dynamic stress cues in the tumor microenvironment could trigger the emergence of clonal expansions with a chromosomal landscape different from the parent tumor cell [205]. Subsequently, these clonal tumor cells acquire spontaneous genetic and epigenetic alterations that can potentially downregulate the *MYC* gene, a pivotal regulator of the epichaperome formation. These developments could result in therapy-resistant clones, as these new clones could harbor mutations that fortify them to evade treatment [200], as illustrated in Figure 3. Moreover, the inherent complexity and dynamic nature of the epichaperome with its many “unknowns,” particularly under varying stress conditions, further complicate and intensify the challenge of piecing together the complete picture of tumor mechanisms. Hence, the challenge is not just in targeting the epichaperome but in designing therapies that factor in the dynamic and adaptive responses thereof.

Going forward, as current targeted treatments may be a great starting point, they must evolve to become truly effective considering the elusive challenges of the epichaperome. Models that incorporate the varying physiological responses of the epichaperome to diverse cues are warranted to gain in-depth insights into the epichaperome biology for better therapy strategies. Furthermore, there will be the need to identify biomarkers that could reveal when tumor cells undergo these reprogramming processes in the epichaperome network. This includes the challenge of how to accurately monitor and understand these dynamic shifts and tailor treatment decisions in real time. Also, innovative targeting strategies that can get therapies directly into tumor cells exhibiting these changes could be a great approach. Equally, the synergy between existing therapies and novel immunotherapies could be explored to complement epichaperome-targeted approaches. Finally, by harnessing the power of AI-driven technologies, treatments could be optimized by determining the timings and types of combinations that effectively combat the dynamic and adaptive nature of the epichaperome.

## 6. Discussion and Perspectives

While our understanding of the epichaperome and the signaling pathways it regulates is growing rapidly, significant challenges remain. Primarily, the complexity of the network due to the vast number of interacting proteins and the intricate regulatory mechanisms poses significant challenges to comprehensive analysis. Another challenge is to map out the spatiotemporal organization in subcellular compartments, owing to the transient nature of PPIs. It is our view and understanding that, given the presence of chaperones and chaperone networks in almost all cellular compartments, an epichaperome-like system is induced in all cellular compartments in stress conditions like cancer. Better still, there could be a control center, likely the cytosol, that connects and modulates the epichaperome-like activities in the remaining cellular compartment. Hence, approaches to piecing together how all these networks consolidate into a whole will be priceless for therapeutic strategies and targeting. Another level of complexity lies in the heterogeneous cell populations and their interactions in tumors. Hence, a map across the cellular subpopulations in different tumors could be equally insightful.

To achieve the above systems biology approach, integrating multiomics data (transcriptomics, proteomics/phosphoproteomics, and metabolomics) to map the full picture and dynamics of the epichaperome network is warranted. Nevertheless, integrating diverse data types (RNA, proteins, and lipids) while maintaining spatial context is technically complicated. Current approaches struggle with sensitivity and spatial resolution limitations, hindering the detection of rare cell types and low-abundance molecules. Overcoming these challenges is vital for advancing insights into cellular interactions, proteomics, and multiomics within tissues.

Additionally, the development of less interfering protein tagging probes [206], high-throughput integrative computational systems, and nanoscale in vivo imaging techniques that make the capture of cue-specific inter- and intracellular epichaperome signaling cascades possible could lead the way to answering many unanswered questions.

Moreover, given the prospects of therapeutically targeting these chaperones, preclinical studies evaluating combined inhibition strategies, potentially alongside other cancer therapies, and the fusion of computational biology to enable efficacy and overcome possible resistance mechanisms will be productive going into clinical trials.

## 7. Conclusions

The epichaperome complex offers a practical approach to strategically combatting cancers. However, the wide possibilities to achieving success in this endeavor require a better understanding of epichaperome biology. Meeting these needs will require improvements in cell biology and proteomics techniques, which cannot be written off. Particularly, single-molecule imaging, enhancements in quantitative multiomics, and protein tagging in non-allosteric and functional domain regions to reduce possible interference in protein activity, and finally, computational biology and AI/machine learning could be revolutionizing.

## Data Availability

The datasets used and/or analyzed during the current study are available from the corresponding author Maxim Shevtsov on reasonable request.

## Abbreviations

AI	Artificial intelligence
BiP	Binding immunoglobulin protein
Cdc37	Cell division cycle 37
Clp	Caseinolytic protease family protein
CSC	Cancer stem cell
DIA	Data-independent acquisition
DYRK3	Dual-specific tyrosine-phosphorylation-regulated kinase 3
ERAD	Endoplasmic reticulum-associated degradation
ERdj3	Endoplasmic reticulum DnaJ3
EMT	Epithelial-to-mesenchymal transition
FKBP52	Immunophillin FK506-binding protein
GRP	Glucose response protein
GSK3β	Glycogen Synthase Kinase-3 Beta
HDA6	Histone deacetylase 6
HIF-1α	Hypoxia Inducible Factor-1α
*HSF*	Heat shock factor
Hsp	Heat shock protein
HSPC	Hematopoietic stem and progenitor Cell
HSR	Heat shock response
IP-MS	Immunoprecipitation–mass spectrometry
LC-MS	Liquid Chromatography–mass spectrometry
LFQ	Label-free quantification
LLPS	Liquid–liquid phase separation
LONP1	LON Peptidase 1
MALDI-TOF MS	Matrix-assisted laser desorption/ionization time-of-flight mass spectrometry
MCPH	Microcephaly primary hereditary
*MYC* gene	Myelocytomatosis oncogene
MPM-ALK	Microphtalmia-associated transcription factor and anaplastic lymphoma kinase
NEF	Nucleotide exchange factor
NudCL2	NudC-like protein 2
NUMA1	Nuclear Mitotic Apparatus Protein-1
PAM	Presequence translocase-associated motor
PML-SYK-fusion	Promyelocytic leukemia gene–spleen tyrosine kinase fusion
PRAR	Peroxisome proliferator–activator receptors
PRM	Parallel reaction monitoring
PTM	Post-translational modification
PU-H71	8-[(6-iodo-1,3-benzodioxol-5-yl)sulfanyl]-9-[3-(propan-2-ylamino)propylpurin-6-amine)
RCC2	Chromosome Condensation 2
SDS-PAGE	Sodium deodecyl sulfate–polyacrylamide gel electrophoresis
SGA	Synthetic genetic array technology
sHsp	Small heat shock proteins
SILAC	Stable isotope labeling by amino acids in cell culture
SYNCRIP	Synaptotagmin-binding cytoplasmic RNA-interacting protein
TCP1	Tailless Complex Polypeptide-1
TGFβ	Transforming growth factor beta
TPR	Tetratricopeptide repeat domain
TNBCs	Triple-negative breast cancer cells
VEGF	Vascular epithelial growth factor
Y2H	Yeast-two hybrid screen
YK5-B	Lysine(K)-specific tryptic-like cleavage site antibody
YK6	Phospho-tyrosine (pTyr-100) monoclonal antibody, clone 6
